# Development of arrival-time diagnostic tool for X-ray pump–probe experiments at Shanghai Soft X-ray Free Electron Laser

**DOI:** 10.1107/S1600577525010653

**Published:** 2026-01-01

**Authors:** Zhi Qiao, Jiadong Fan, Zichen Gao, Yonggan Nie, Pingping Wen, Xinyuan Wang, Kun Yan, Hang Ren, Boyong Wang, Jiaming Jiang, Yuneng Shen, Yongxing Zhang, Wenjing Zhu, Guan Shu, Chaofan Xue, Zhi Guo, Zipeng Liu, Hanxiang Yang, Zheng Qi, Kaiqing Zhang, Tao Liu, Zhen Wang, Chao Feng, Yajun Tong, Zhi Liu, Huaidong Jiang

**Affiliations:** a Center for Transformative Science ShanghaiTech University Shanghai China; b Shanghai Advanced Research Institute Chinese Academy of Sciences Shanghai China; c School of Physical Science and Technology ShanghaiTech University Shanghai China; University College London, United Kingdom

**Keywords:** X-ray pump/probe experiments, X-ray free electron lasers, timing diagnostics, femtosecond lasers

## Abstract

X-ray free electron lasers (XFELs) serve as advanced light sources and have become essential for investigating ultrafast dynamic phenomena in physics and materials with extraordinary resolution. Owing to the XFEL’s ultrafast characteristics and short wavelengths, an arrival-timing tool is crucial for pump–probe experiments. To address this, we have developed a timing diagnostic tool employing both spectral-encoding and spatial-imaging methods at the SBP beamline for the newly constructed Shanghai Soft X-ray Free Electron Laser Facility (SXFEL). This timing tool was experimentally validated, proving that the spectral-encoding technique could achieve single-pulse measurement with an accuracy of under 40 fs [root mean square (RMS)], and exhibited a timing-jitter measurement of 90.3 fs (RMS) at the CSI endstation of SXFEL. Furthermore, the spatial-imaging approach used both polished- and rough-surface GaAs crystals, which simplifies implementation in X-ray pump–probe experiments, and allows for the characterization of X-ray pulse arrival times at the endstation without rotating the sample stage. These findings confirm that the timing diagnostic tool provides dependable high-precision temporal characterization of X-ray pulses at SXFEL, facilitating high-accuracy X-ray pump–probe experiments.

## Abstract 2

The development of a timing diagnostic tool at the CSI endstation of the Shanghai Soft X-ray Free Electron Laser Facility achieves precise temporal characterization for advanced pump–probe experiments. The tool’s unique design enables X-ray pulse monitoring without sample-stage rotation with both spectral-encoding and spatial-coupling methods, offering a reliable solution for X-ray pump–probe experiments at the CSI endstation.

## 1. Introduction

With the advancement of ultrafast lasers, it has become possible to characterize material dynamics on timescales ranging from femtoseconds to even attoseconds. Unlike conventional optical pump–probe and electron diffraction techniques, ultrafast X-ray pulses generated by X-ray free electron lasers (XFELs) based on self-amplified spontaneous emission (SASE) enable real-time probing of structural dynamics in bulk materials with a temporal resolution of tens of femtoseconds or even attoseconds (Barty *et al.*, 2013 ▸; Chapman *et al.*, 2006 ▸; Huang *et al.*, 2021 ▸; Pellegrini *et al.*, 2016 ▸), considering the deeper penetration of X-rays. Furthermore, the high peak brilliance and coherence of these X-rays have paved the way for high-resolution X-ray scattering, diffraction and spectroscopic methods, emphasizing the significance of ultrafast characterization of dynamic processes induced by ultrafast optical lasers, commonly referred to as the pump–probe technique (Lemke *et al.*, 2013 ▸; Inoue *et al.*, 2016 ▸; Guo *et al.*, 2024 ▸; Bergmann *et al.*, 2021 ▸; Allaria *et al.*, 2013 ▸).

Unlike the pump–probe technique of optical lasers, where the pump laser and probe laser are generally generated from the same source, and thus the timing of the pump and probe lasers is inherently synchronized, a complicated timing synchronization system is required to synchronize the X-ray pulse from the electron accelerator and the optical femtosecond pulse from a normal laser cavity (Kang *et al.*, 2017 ▸). With the synchronization system, a shot-to-shot time jitter of less than 100 fs between the optical laser pulse and the X-ray pulse can be achieved. However, accurately determining and monitoring the relative delay between the XFEL pulse and the optical laser, with an accuracy of less than the time jitter at the endstation, is essential to measure the timing drift and the arrival time for the pump–probe experiment, thus ensuring precise measurement of ultrafast dynamics, for example, the breaking and forming of chemical bonds, ultrafast phase transitions, charge transfer, and damage (Barends *et al.*, 2024 ▸; Chapman *et al.*, 2014 ▸; Kim *et al.*, 2022 ▸). However, measuring the arrival time of the X-ray pulse is much more difficult than measuring that of optical lasers because of the short X-ray wavelength and the low-interaction cross section between X-rays and matter. To measure the arrival time of the XFEL pulse relative to the optical laser pulse, several methods have been developed (Meyer *et al.*, 2006 ▸; Azima *et al.*, 2009 ▸; Fritz *et al.*, 2007 ▸; Harmand *et al.*, 2013 ▸; Durbin *et al.*, 2012 ▸; Eckert *et al.*, 2015 ▸; Ivanov *et al.*, 2018 ▸), which are mainly based on either the terahertz beam or the X-ray-induced optical reflectivity/transmission changes in materials. Due to the complexity and cost of the terahertz streaking technique, the X-ray-induced optical reflectivity/transmission change is commonly used in FEL facilities for arrival-time monitoring (Bionta *et al.*, 2011 ▸; Maltezopoulos *et al.*, 2008 ▸; Gahl *et al.*, 2008 ▸; Schorb *et al.*, 2012 ▸; Beye *et al.*, 2012 ▸).

The change in free carrier density induced by the absorbed X-ray fluence causes a rapid shift in refractive indices through photoionization and subsequent cascade ionization, resulting in a quick change in surface reflectivity and bulk transmission (Durbin *et al.*, 2012 ▸; Durbin, 2012 ▸). Materials such as GaAs, YAG and silicon nitride are typically used for high temporal resolution measurements of XFEL pulse arrival times by detecting reflected or transmitted optical laser signals with a photodiode or imaging CCD. Arrival-time diagnostics for refractive-index change commonly employ three techniques: spatial imaging, spatial encoding and spectral encoding. Both spatial and spectral encoding offer the significant advantage of enabling single-shot measurements. However, each presents challenges for implementation directly at the sample position. The spatial-encoding technique, for instance, requires a large X-ray spot size to map the time delay onto a spatial coordinate, thereby covering a wide temporal range. This large spot necessitates a correspondingly high X-ray pulse energy to induce a detectable change in the refractive index. Similarly, the spectral-encoding method demands precise spatial overlap and matching beam sizes between the femtosecond optical laser and the X-ray beam to achieve a high signal-to-noise ratio in the resulting spectral modulation. These requirements are fundamentally at odds with the typical experimental conditions at the endstation. For techniques such as X-ray scattering or diffraction, the X-ray beam is focused to a micrometre-scale spot at the sample position to provide high flux density. Consequently, the constraints of both spatial and spectral encoding render them unsuitable for arrival-time diagnostics at the sample position of the experiment. In contrast, the spatial-imaging method is compatible with measurements at the sample position, but it is a scanning method that requires multiple shots to determine the arrival time. In addition, the off-axis long-working-distance microscopes typically used at endstations possess a spatial resolution that is often insufficient to resolve the micrometre-sized interaction area of the focused FEL beam.

To address this challenge, we have developed timing diagnostic tools that combine spectral encoding for single-pulse measurements with spatial imaging using a coaxial microscope with high spatial resolution. This approach offers superior spatial resolution, facilitating the precise determination of the FEL pulse arrival time in X-ray pump–probe experiments conducted at the SBP beamline of the Shanghai Soft X-ray Free Electron Laser Facility (SXFEL) (Fan *et al.*, 2022 ▸). SXFEL has recently commenced user experiments; however, a crucial component for pump–probe studies is an arrival-time diagnostic tool capable of accurately characterizing the time delay between the XFEL pulse and the femtosecond laser pulse, with a precision surpassing the time jitter of the XFEL beam. According to the current structure of SXFEL, the time jitter between the XFEL pulse and the femtosecond laser is estimated to be around 100 fs; therefore, this arrival-time diagnostic tool is designed to provide a measurement accuracy better than 100 fs.

The spectral-encoding method is widely used to measure the arrival time of X-ray pulses, and has demonstrated excellent performance in single-pulse timing-jitter measurements. Consequently, the spectral-encoding method, by chirping the femtosecond pulse temporally, is chosen for single-pulse timing diagnostics at the SBP beamline of SXFEL. By employing spectral encoding, we achieved single-pulse arrival-time monitoring for the X-ray pulse. To realize the arrival-time measurement at the endstation for X-ray pump–probe experiments, a spatial-imaging method is also employed, using a coaxial inline microscope with a high magnification of 10× to observe reflectivity changes in the pump femtosecond laser induced by the XFEL pulse by scanning the time delay of the femtosecond laser. We compared the spatial-imaging technique using both rough and polished GaAs surfaces as the sample, demonstrating that the relative delay between the FEL and optical lasers could be accurately determined and monitored under the same X-ray pump–probe experimental conditions. Using these timing diagnostic tools at SXFEL, we successfully synchronized the X-ray and optical laser pulses for the pump–probe experiment.

## 2. Methods and experimental setup

**Figure 1:**
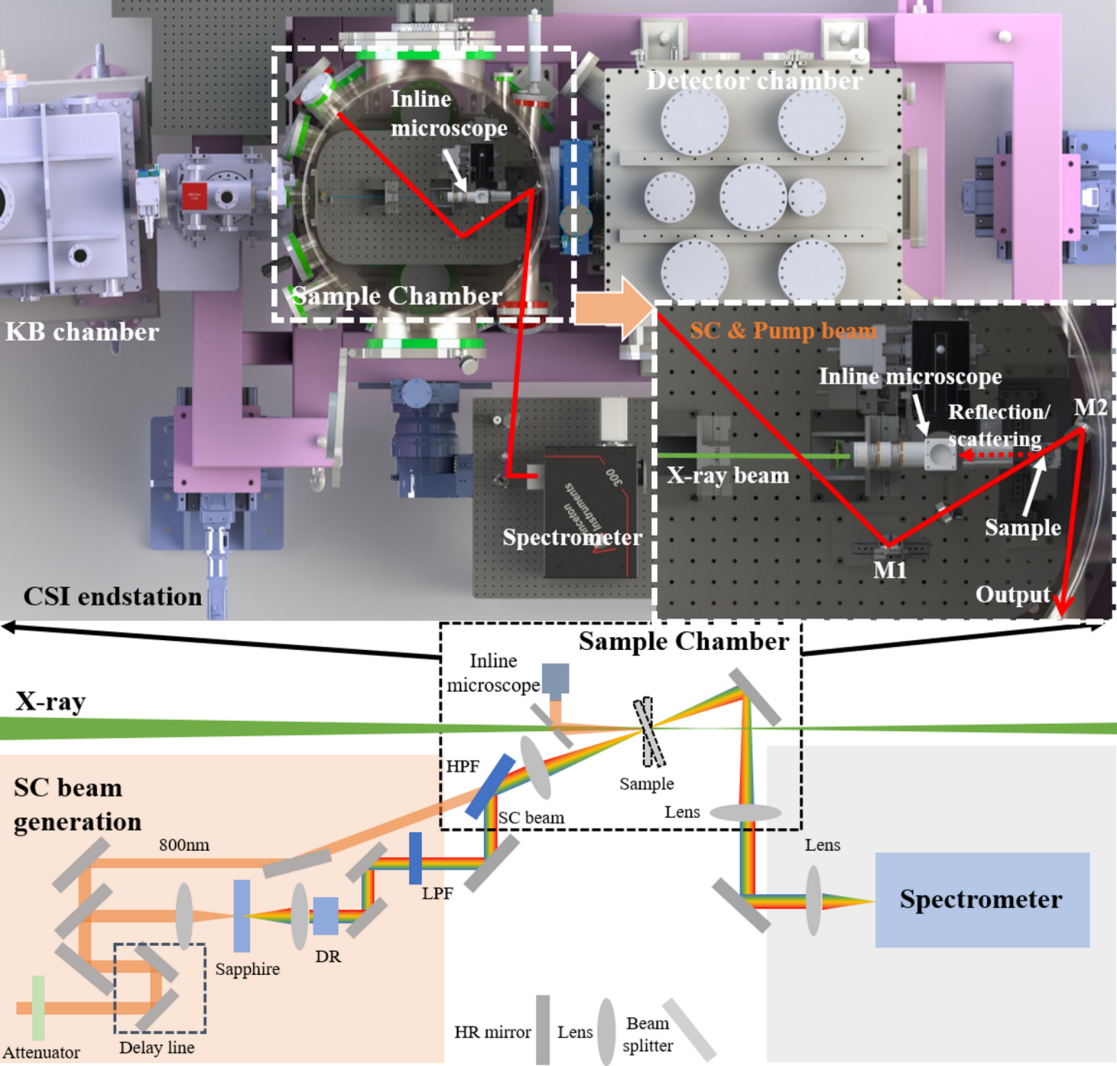
Schematic drawings of the timing diagnostic tool at the CSI endstation of the SPB beamline at SXFEL. The top figure shows the layout of the CSI endstation with a zoomed-in sample chamber. The bottom figure shows the setup of the arrival-time tool, where the transmitted beam from the sample is reflected into the spectrometer for the spectral-encoding method and the reflected or scattered beam is acquired by the inline microscope for the spatial-imaging method. (The red line shows the femtosecond laser and the SC beam; the green line shows the X-ray beam; LPF: low-pass filter; HPF: high-pass filter; DR: dispersion rod; M1: high-reflection mirror in sample chamber; M2: high-reflection mirror in sample chamber.)

The setup of the timing diagnostic tools, incorporating the previously mentioned spectral-encoding and spatial-imaging methods, is integrated into the Coherent Scattering and Imaging (CSI) endstation, as shown in Fig. 1 ▸. The Ti:sapphire femtosecond laser, comprising a mode-locking oscillator and a regenerative amplifier, is synchronized with the injection laser of the accelerator with a jitter of less than 10 fs. It generates a pulse width of 40 fs with a central wavelength of 800 nm. The process of generating the supercontinuum (SC) beam used in the spectral-encoding method is illustrated in the lower schematic drawing of Fig. 1 ▸, and the laser parameters are shown in Table 1 ▸. A fraction of 10% of the femtosecond laser is used for the generation of SC (white beam), where the adjustable attenuator is inserted to control the pulse energy on the sapphire plate with a thickness of 1 mm, while 90% of the femtosecond laser serves as the experiment’s pump laser. Using a high-pass filter, the pump laser is made collinear with the SC beam. The pump laser with a wavelength of 800 nm was used for the spatial-imaging method of the timing tool. The focal length of the lens in front of the sapphire plate is 100 mm, providing a tight focus. An achromatic lens with a 100 mm focal length is then used to collimate the SC beam. For precise timing adjustments, the delay line consists of a long-travel linear stage (Newport Corp) and 90° dual mirrors. A 10 mm-long dispersion rod (TIH53) is employed to temporally stretch the SC, and a low-pass filter is inserted to eliminate any residual pump laser from the SC beam. To match the beam size of the femtosecond laser with the XFEL beam, an achromatic lens with a 150 mm focal length is used to focus the SC beam to 30 µm [full width at half-maximum (FWHM)]. As shown in Fig. 1 ▸, the SC beam, or the pump beam, is reflected by the M1 mirror inside the sample chamber to the sample, and the transmitted beam is reflected by the M2 mirror outside the chamber to the spectrometer (Princeton Instruments, HRS300) with a grating of 300 lines mm^−1^ and a CMOS camera (Andor, Zyla 5.5) when transparent samples such as YAG and silicon nitride are used, and the spectral encoding is implemented. For the spatial-imaging method, the reflected beam or the scattered beam from the sample, such as GaAs, is acquired by the high-resolution inline microscope. The femtosecond laser without SC generation is used as the pump beam for the pump–probe experiment as shown in Fig. 1 ▸. A portion of 10% of the femtosecond laser beam is used for SC generation, which has a maximal pulse energy of 1 mJ, but the pulse energy of the femtosecond laser used must be attenuated to ∼1 µJ to avoid damage to the sapphire plate.

The X-ray beam is generated through the SASE process and focused by a pair of Kirkpatrick–Baez mirrors to a size of ∼6 µm (FWHM). In order to increase the interaction efficiency on the sample for arrival-time diagnostics for the spectral-encoding case, the sample is moved upstream to get a defocused X-ray beam size of 10 µm considering the femtosecond-laser beam size of 30 µm. For comparison, the sample is moved back to the focal-spot position for the spatial-imaging method, where the coaxial inline microscope is implemented to monitor the reflected pump beam from the sample when excited by the X-ray beam. As shown in Fig. 1 ▸, the inline microscope with a magnification of 10× was used to monitor the reflectivity variation when the sample was excited by the X-ray pulse. The reflected femtosecond-laser beam was imaged with a spatial resolution of 1.5 µm and an effective pixel size of 340 nm when the sample was illuminated by the laser beam with an angle of 30°. For the spectral-encoding method, a YAG sample with a thickness of 100 µm and silicon nitride (Si_3_N_4_) windows with a thickness of 200 nm are used, while the GaAs sample is used for the spatial method. A specially designed mechanical mount with an angle of 15° is needed to reflect the laser beam into the coaxial microscope for the case of polished GaAs sample, while it is not necessary for the case of GaAs with a rough surface as shown below, which extends the robustness and simplifies the setup.

## 3. Experimental results

### 3.1. Spectral-encoding method

**Figure 2:**
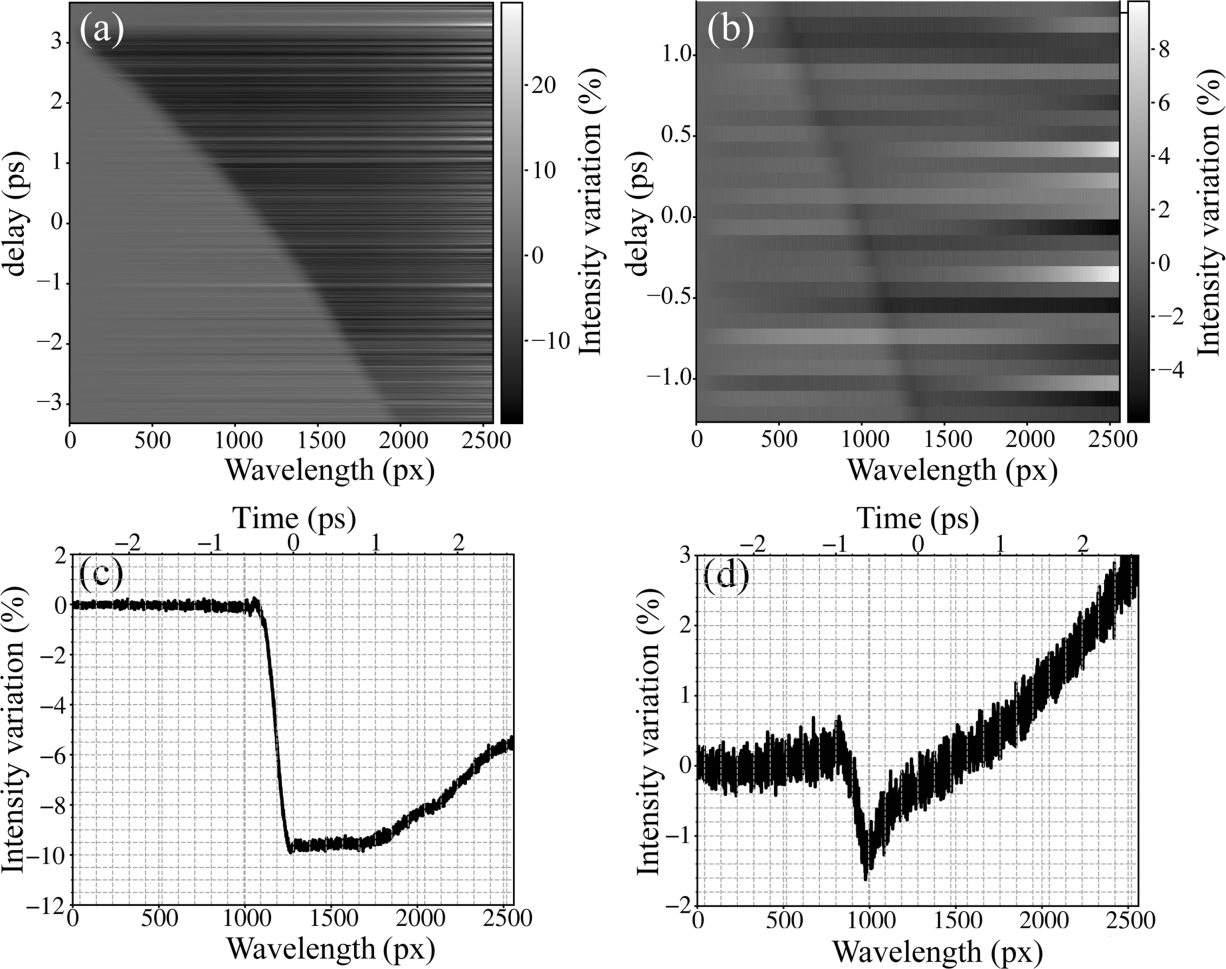
Spectral-encoding timing diagnostics of XFEL pulses. (*a*) Spectrum versus SC beam delay time for a YAG sample. (*b*) Spectrum versus SC beam delay for a silicon nitride sample. (*c*) Spectrum line profile at a delay of 0 for a YAG sample. (*d*) Spectrum line profile at a delay of 0 for a silicon nitride sample.

The SBP beamline of SXFEL is running at 10 Hz with an X-ray energy of 420 eV and a single-pulse energy of around 10 µJ at the focus point. The spectral-encoding method is first verified to measure the timing properties of the single XFEL pulse. Fig. 2 ▸ shows the spectrum variation while changing the delay time between the XFEL pulse and the SC beam. Both the YAG sample and the silicon nitride window are used to measure the X-ray induced transmission change. Since a dispersion rod of 10 mm is used, the temporal window of the stretched SC pulse is around 6 ps for a spectrometer with a grating of 300 lines mm^−1^. Due to the nonlinear dispersion of the TIH53 glass, the reflectivity change of the spectrum shows a nonlinear dependence on the wavelength while scanning the relative time delay as shown in Fig. 2 ▸(*a*). Figs. 2 ▸(*c*) and 2 ▸(*d*) show the spectrum change with an average of 100 pulses after removing background at the time delay of 0 ps, which shows that the transmission change of the YAG sample could be around 10%, while it is around 2% for the silicon nitride window. The high modulation induced by the X-ray pulse of the YAG sample is caused by the higher absorption due to the large thickness and intrinsic response of the material. During the measurement, the XFEL pulse energy fluctuates due to the SASE process, so the observed transmission change may diminish or even disappear when scanning the delay time.

**Figure 3:**
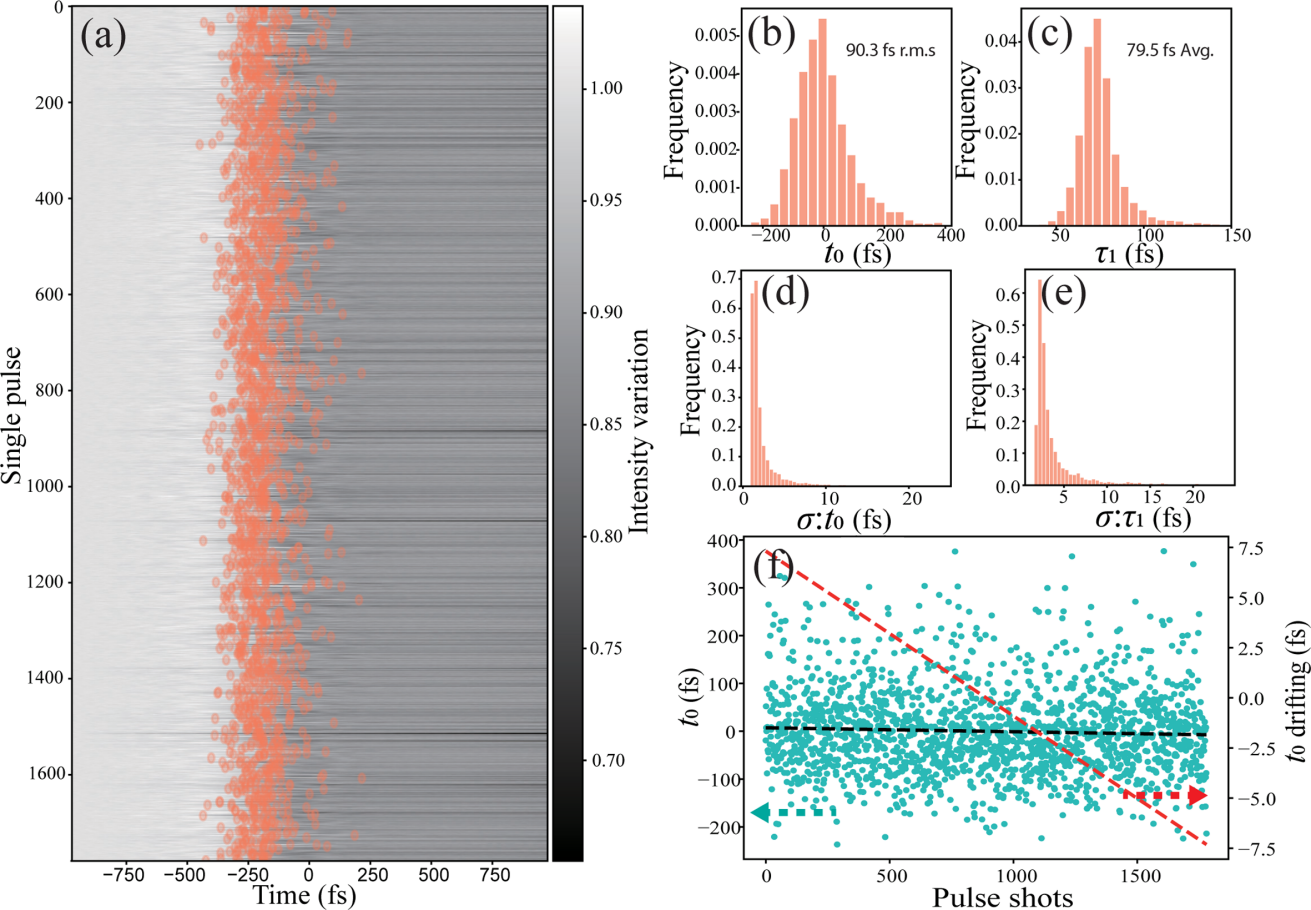
Arrival time of a single FEL pulse using the spectral-encoding timing diagnostic tool. (*a*) Spectrum of a single FEL pulse. (*b*) Distribution of the arrival time of a single pulse. (*c*) Distribution of the falling edge of a single pulse. (*d*) Distribution of the uncertainty of the arrival time *t*_0_. (*e*) Distribution of the uncertainty of the falling edge τ_1_. (*f*) Arrival time of 1800 pulses (the black dashed line represents the linear fit of the arrival time of each pulse and the red dashed line shows the enlarged linear fit on the right *y* axis, illustrating the drift of the XFEL pulses).

One of the advantages of the spectral-encoding method is the single-pulse measurement capability. Here we used spectral encoding of the YAG sample to monitor the timing characteristics of the XFEL beam, which are shown in Fig. 3 ▸. A total of 5000 pulses were acquired at a repetition rate of 10 Hz, and 1800 pulses were selected with pulse energies above the threshold, which provides effective modulation of the spectrum and is monitored by the gas monitor detector (GMD), as shown in Fig. 3 ▸(*a*). For each single-pulse spectrum line profile, as shown in Fig. 2 ▸(*c*), the temporal property is obtained by fitting the spectrum with the commonly used equation
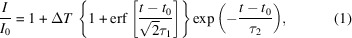
where Δ*T* is the maximum transmission change, *t*_0_ is the fitted arrival time of the XFEL pulse, τ_1_ represents the falling edge of the erf function related to the intrinsic properties of the materials and τ_2_ is the exponential decay of the transmission change. As shown in Fig. 3 ▸(*a*), the arrival time *t*_0_ is the orange circles, showing the pulse-by-pulse variation along the spectrum. Since the grating of 300 lines mm^−1^ is used and the SC pulse is stretched to 6 ps, the temporal resolution corresponding to the wavelength is 2.1 fs pixel^−1^ and can be obtained from the delay scanning curve in Fig. 2 ▸(*a*), and the statistics of the fitted arrival time and falling edge are shown in Figs. 3 ▸(*b*) and 3 ▸(*c*). After monitoring the 1800 pulses, the timing jitter [root mean square (RMS)] of the arrival time is 90.3 fs, as shown in Fig. 3 ▸(*b*), which provides the timing synchronization accuracy between the femtosecond laser and the XFEL pulses. From the fitted falling edge of the spectrum change of each single pulse, the falling edge τ_1_ gives a mean value of 79.5 fs (RMS) and a variation of 31 fs (RMS) corresponding to a Gaussian function width of 187 fs (FWHM). Since the falling edge is determined by the intrinsic response of materials, and the XFEL and laser pulse width, the XFEL pulse width can be estimated to be (187^2^ − 150^2^)^1/2^ = 111 fs (FWHM) with a YAG response of 150 fs, which matches the parameters of the accelerators. Since the arrival time and falling edge are dependent on the curve-fitting process, the fitting uncertainty is calculated for each pulse and gives an averaged uncertainty of 2.47 fs and 3.8 fs for the fitting of *t*_0_ and τ_1_ as shown in Figs. 3 ▸(*d*) and 3 ▸(*e*), respectively. Considering that the fit τ_1_ is affected by the intrinsic response of materials, the XFEL pulse duration and the temporal resolution of the measurement, the variation of the fitted falling edge provides a clue to characterize the temporal resolution of the setup. As mentioned above, the falling edge τ_1_ is within a range of 187 ± 3.8, which sets an upper limit for the temporal resolution to the error of the fits *T*_res_ ≤ [(187 + 3.8)^2^ − 111^2^ − 150^2^]^1/2^ ≃ 40 fs (Schorb *et al.*, 2012 ▸). The variation of the fitted falling edge also proves that the measurement accuracy is at a similar level. The variation of τ_1_ with multiple measurements is 31 fs, which is mainly induced by the measurement accuracy or the temporal resolution of the setup. In the spectral-encoding measurement, the spectrometer with a grating density of 300 lines mm^−1^ was used, giving a dispersion of 10.4 nm mm^−1^, and an optical spectral resolution of 0.4 nm corresponding to a temporal resolution of 12.4 fs. Due to the systematic error of the setup, the temporal resolution is slightly larger than the ideal optical resolution of the spectrometer.

The arrival time of the XFEL beam is monitored for 5000 pulses by the spectral-encoding timing tool, and there exists obvious drifting between the femtosecond laser and the XFEL pulse, which is shown in Fig. 3 ▸(*f*). The black dashed line is the linear fitting of the selected 1800 pulses with high pulse energy measured by the GMD, and the red dashed line is the enlarged figure of the linear fitting, from which the *t*_0_ drifting is around 15 fs during the acquisition of the 5000 pulses (although the machine is running at 10 Hz repetition rate, the data acquisition was a bit slower than 10 Hz). The arrival-time drifting attributes the environmental variation, and proves that the online monitoring of arrival time is essential for experiments requiring high-accuracy timing.

### 3.2. Spatial-imaging method

Although the spectral-encoding method can provide high-accuracy single-pulse measurements, it is difficult to integrate the diagnostic tool with the experimental setup simultaneously because the YAG crystal or silicon nitride window was placed at a defocused position where the XFEL beam size matched the SC beam size to achieve a high signal-to-noise ratio in the spectral modulation. Therefore, the spectral-encoding method could not measure the exact arrival time at the sample position where the pump–probe experiment was conducted. Here we combine the spatial-imaging method and the timing diagnostic tool with the spectral-encoding method, showing that the spatial-imaging method can provide accurate arrival-time measurements during X-ray pump–probe experiments. The timing tool operates invasively, so the spectral encoding is used to characterize the time jitter of the XFEL machine, while the spatial-imaging method is used to determine the arrival time before starting the pump–probe experiment. A [100] GaAs sample was used for the arrival-time measurement. Due to the free carrier density induced by photoionization and ionization when the X-ray illuminates the sample, the refractive index of GaAs rapidly changes, resulting in a fast reflectivity and transmission change within less than 100 fs. By monitoring the reflectivity or transmission change of the laser beam, the arrival time of the X-ray pulse can be determined. Considering that the reflectivity change induced by the X-ray pulse is relatively weak, an X-ray shutter was implemented so that the reflected image without the X-ray beam was also acquired for background correction, reducing the data-acquisition rate to 5 Hz. The relative delay between the femtosecond laser and the X-ray pulse was tuned by the delay line within a range of 10 ps, and the reflectivity map at each time delay was analyzed to identify the decremental jump.

**Figure 4:**
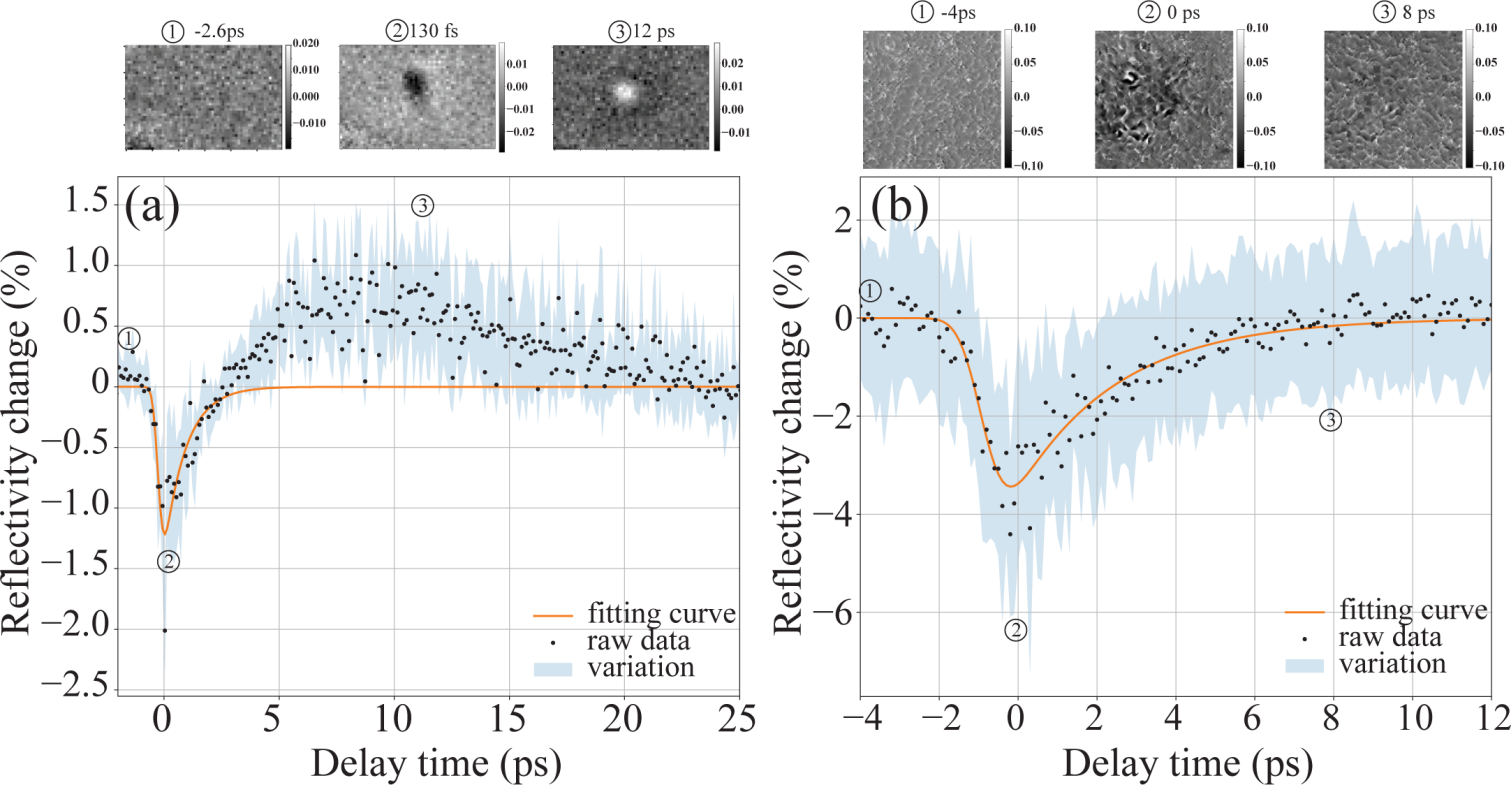
Arrival-time diagnostics by spatial coupling of GaAs. (*a*) Laser-reflectivity variation versus delay time between the FEL and laser pulse using a polished smooth surface. (*b*) Laser-reflectivity variation versus delay time between the FEL and laser pulse using a surface roughened with P7000 sandpaper.

Since the femtosecond laser and X-ray beam are off-axis, the sample, such as GaAs, has to be rotated by an angle to direct the reflected laser beam into the inline microscope, which affects the X-ray pump–probe experimental setup. Instead of using polished GaAs crystal, we demonstrated that GaAs crystal with a rough surface polished by sandpaper offers higher flexibility to the experimental setup, and no angle rotation is needed. Fig. 4 ▸ shows the measured reflectivity variation curve along with the delay time between the femtosecond laser and the XFEL pulse using both the polished GaAs crystal and the rough-surface GaAs crystal. As previously mentioned, a specially designed sample mount was used so that the crystal was rotated by an angle of 15° for the polished surface, while the GaAs crystal was mounted on the sample holder without rotation for the rough-surface case. At each time delay, the measurement was repeated 50 times, and in Fig. 4 ▸ the black dots and light blue areas show the mean and variation (RMS) of the reflectivity, respectively. Then, the curve was fitted by equation (1) to give the arrival time and timing jitter.

For the polished surface, the measured reflectivity curve gives a fitted falling edge of 185 fs (RMS), which is larger than the obtained τ_1_ of the spectral-encoding case due to the timing jitter of the X-ray pulse and laser pulse. The arrival-time fitting uncertainty of the reflectivity curve is 2.8 fs, which demonstrates that the spatial-imaging method gives good accuracy for the measurement of the arrival time. Different from the sample of YAG, the reflectivity variation of GaAs increases after 3 ps, excited by the X-ray pulse, as shown in Fig. 4 ▸(*a*). Compared with the polished-surface case, the GaAs crystal with the rough surface can simplify the alignment process and the experimental setup. As shown in Fig. 4 ▸(*b*), the reflectivity curve gives obvious speckle-like modulation in the acquired images, and the calculated reflectivity modulation is higher because of better background subtraction. The uncertainty of the fitting process is also 2.7 fs, similar to the smooth-surface case. The rough-surface case was measured with a different machine status when the XFEL was run at an energy of 124 eV, therefore the fitted falling edge is 470 fs, which is larger than the value of the polished-surface case. Compared with the spectral-encoding method, the spatial-coupling method shows higher noise and lower accuracy due to the fluctuation of the XFEL beam position and pulse energy. The spatial-imaging method has advantages in the implementation of pump–probe experiments since the sample, such as GaAs, can be mounted with the experimental sample together at the same position. In addition, it is not necessary to rotate the sample stage by an angle to reflect the laser beam into the inline objective with the rough surface, since the scattering of the laser beam is sufficient for the inline microscope.

## 4. Conclusions

In conclusion, a timing diagnostic tool was developed and tested at the SBP beamline of SXFEL, incorporating spectral-encoding and spatial-imaging methods. The spectral-encoding method gives a temporal resolution better than 40 fs, and the timing jitter between the X-ray pulse and the femtosecond laser was measured to be 90.3 fs (RMS) for single pulses at the CSI endstation of the SXFEL SBP beamline. For the spatial-imaging method, both polished- and rough-surface GaAs crystals are used. By employing a GaAs crystal with a rough surface, *in situ* measurements of the X-ray pulse arrival time during pump–probe experiments were successfully performed without altering the rotation angle of the sample stage. The timing diagnostic tool provides reliable high-resolution measurements of the temporal characteristics of X-ray pulses at the SXFEL CSI endstation, facilitating high-accuracy X-ray pump–probe experiments.

## Figures and Tables

**Table 1:** Beam parameters of the XFEL and femtosecond laser

Parameter	XFEL beam	Femtosecond laser
Energy/wavelength	100–620 eV	800 nm
Pulse energy	∼50 µJ	∼10 mJ
Pulse duration (FWHM)	≤100 fs	40 fs
Focal size (FWHM)	∼6 µm	∼30 µm

## Data Availability

Data underlying the results presented in this paper are not publicly available but may be obtained from the authors upon reasonable request.
